# Supplementary material for: Visualizing looping of two endogenous genomic loci using synthetic zinc‐finger proteins with anti‐FLAG and anti‐HA frankenbodies in living cells
Source: Genes Cells. 2021 Sep 20;26(11):905–26. doi: 10.1111/gtc.12893 (PMC8893316; doi:10.1111/gtc.12893)
Supplement: Supplementary file 1 — Fig S1‐S7 [file GTC-26-905-s002.pdf]

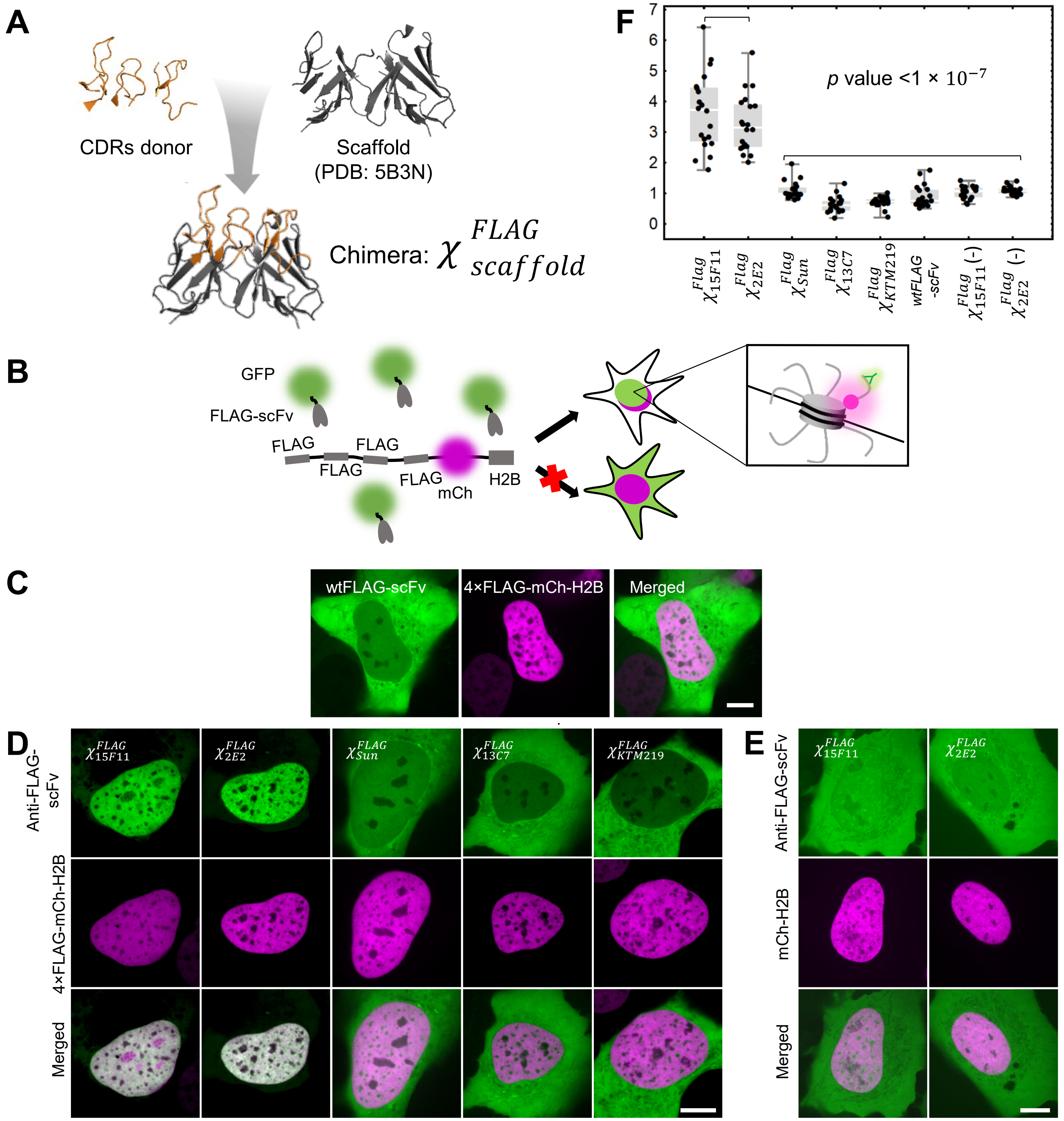

Fig. S1. Design strategy and initial screening of anti-FLAG frankenbodies.
(**A**) A cartoon schematic showing how to design a chimeric anti-FLAG scFv using wtFLAG-scFv CDRs and stable scFv scaffolds. (**B**) A cartoon showing how to screen the five chimeric anti-FLAG scFvs in living U2OS cells. (**C**)A representative cell showing the respective localization of the wildtype anti-FLAG-scFv in living U2OS cells co-expressing FLAG-tagged histone H2B (wtFLAG-scFv, green; 4 × FLAG-mCh-H2B, magenta). (**D**) Initial screening results showing the respective localization of the five chimeric anti-FLAG scFvs in living U2OS cells co-expressing FLAG-tagged histone H2B (chimeric anti-FLAG scFv, green; 4 × FLAG-mCh-H2B, magenta). (**E**) Control results showing the respective localization of anti-FLAG frankenbodies in living cells lacking FLAG-tagged histone H2B (chimeric anti-FLAG scFv, green; mCh-H2B, magenta). (**F**) Nuclear to cytoplasmic fluorescent intensity ratio (Nuc/Cyt) plot of each chimeric anti-FLAG scFv and wtFLAG-scFv for all cells imaged as in (**C**), (**D**) and (**E**). Mann-Whitney test. All images are representative cell images from one independent experiment. Scale bars: 10μm. Source data are provided as a Source Data file. For box plots, center lines show the medians; the boxes indicate 25-75%; whiskers extend 1.5 times the interquartile range from the 25th and 75th percentiles; and data points are plotted.

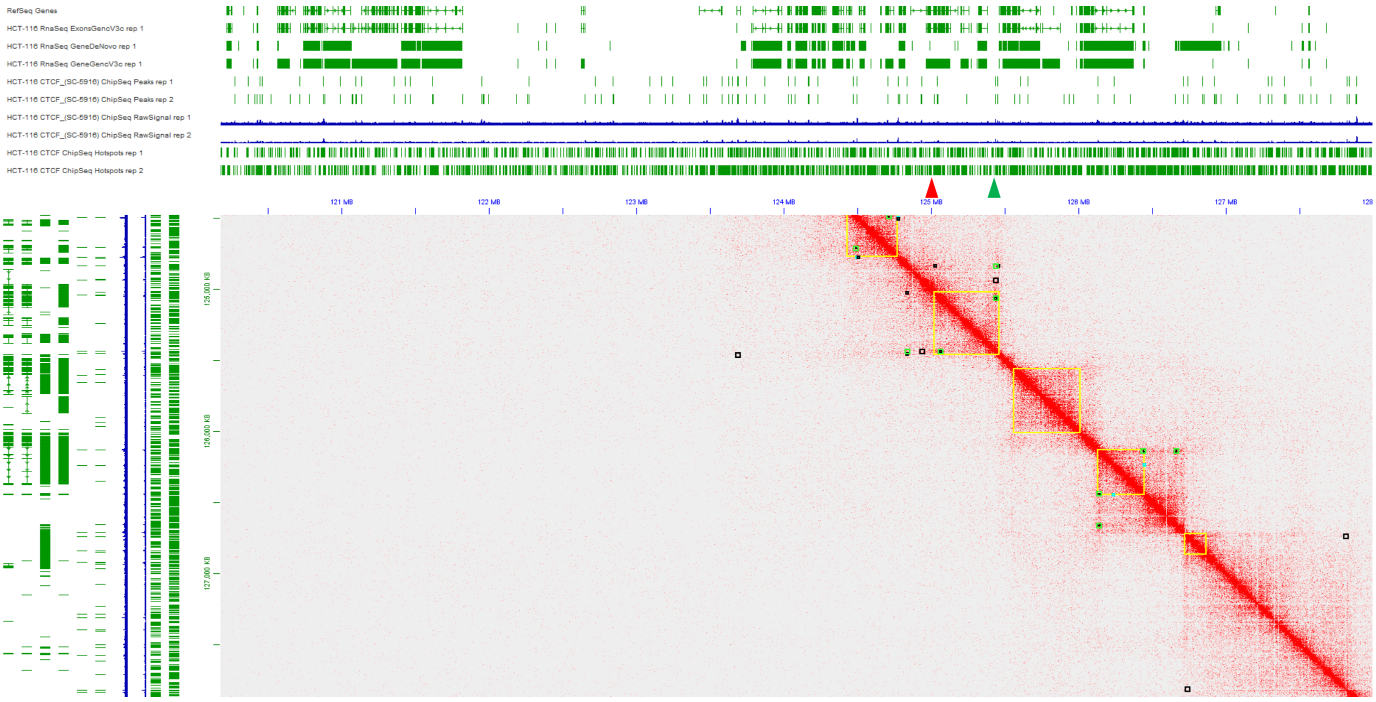

TAD: chr8:126,150,000-126,750,000

▲ ZF probe F
>gi|568815590|ref|NC_000008.11|:126151180-126151433 Homo sapiens chromosome 8, GRCh38.p7 Primary Assembly
AGC ATT CTT AA G GGA AAG AAA TTC CAG CCA AGA TTT TCA TGT ACC ATC AAA TGA AGC TTC

▲ ZF probe R
>gi|568815590|ref|NC_000008.11|:126711633-126712058 Homo sapiens chromosome 8, GRCh38.p7 Primary Assembly
GGG GGA ATC TCC TGA CAC AGG CCC TAG CTG TGA GGG TGG AGA GAA GTT TTA CTT TTT AAT

Fig. S2. ZF probe target sites.
Chromatin contact sites identified by multiple HiC algorithms, HiC pro (Black), Juicebox(Green), and data from Li et al, 2012 (Yellow) is aligned with ENCODEHCT116 CTCF ChipSeq data (ENCSR000BSE) and RNASeq (ENCSR000CWM) data. ZF probes that bind to the upstream and downstream sequence (underlined) are named F and R, respectively.

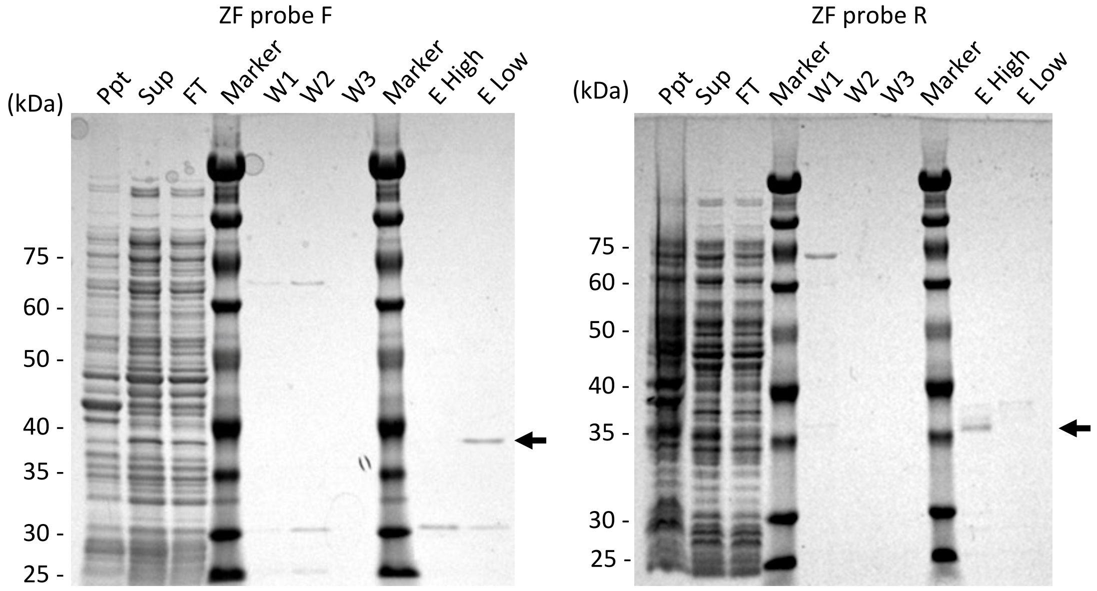

LEPGEKPYKCPECGKSFS**QRANLRA**
HQRTHTGEKPYKCPECGKSFS**RKDNLKN**
HQRTHTGEKPYKCPECGKSFS**QRAHLER**
HQRTHTGEKPYKCPECGKSFS**RKDNLKN**
HQRTHTGEKPYKCPECGKSFS**TTGALTE**
HQRTHTGEKPYKCPECGKSFS**HKNALQN**
HQRTHTGKKTS
+10x HA tag

LEPGEKPYKCPECGKSFS**QLAHLRA**
HQRTHTGEKPYKCPECGKSFS**DPGHLVR**
HQRTHTGEKPYKCPECGKSFS**RSDELVR**
HQRTHTGEKPYKCPECGKSFS**QRANLRA**
HQRTHTGEKPYKCPECGKSFS**TTGALTE**
HQRTHTGEKPYKCPECGKSFS**RSDHLTN**
HQRTHTGKKTS
+10x Flag tag

Fig. S3. Expression and purification of ZF probes.
Lysates of *E. coli* expressing ZF probes were centrifuged to remove the insoluble precipitate (Ppt). The supernatant (Sup) was applied to an Ni column and the flow-through (FL), Wash 1 (W1), Wash 2 (W2), Wash 3 (W3) fractions were collected. Bound proteins were then eluted and the elution peak 1(E high) and peak 2 (E low) were collected. All fractions were analyzed by SDS-PAGE and Coomassie Blue staining. The sizes of marker proteins (Marker) are indicated on the left. The positions of ZF probes are indicated by arrows on the right. Amino acid sequence of ZF proteins are shown with the variable regions that determine the DNA-binding specificity indicated in bold.

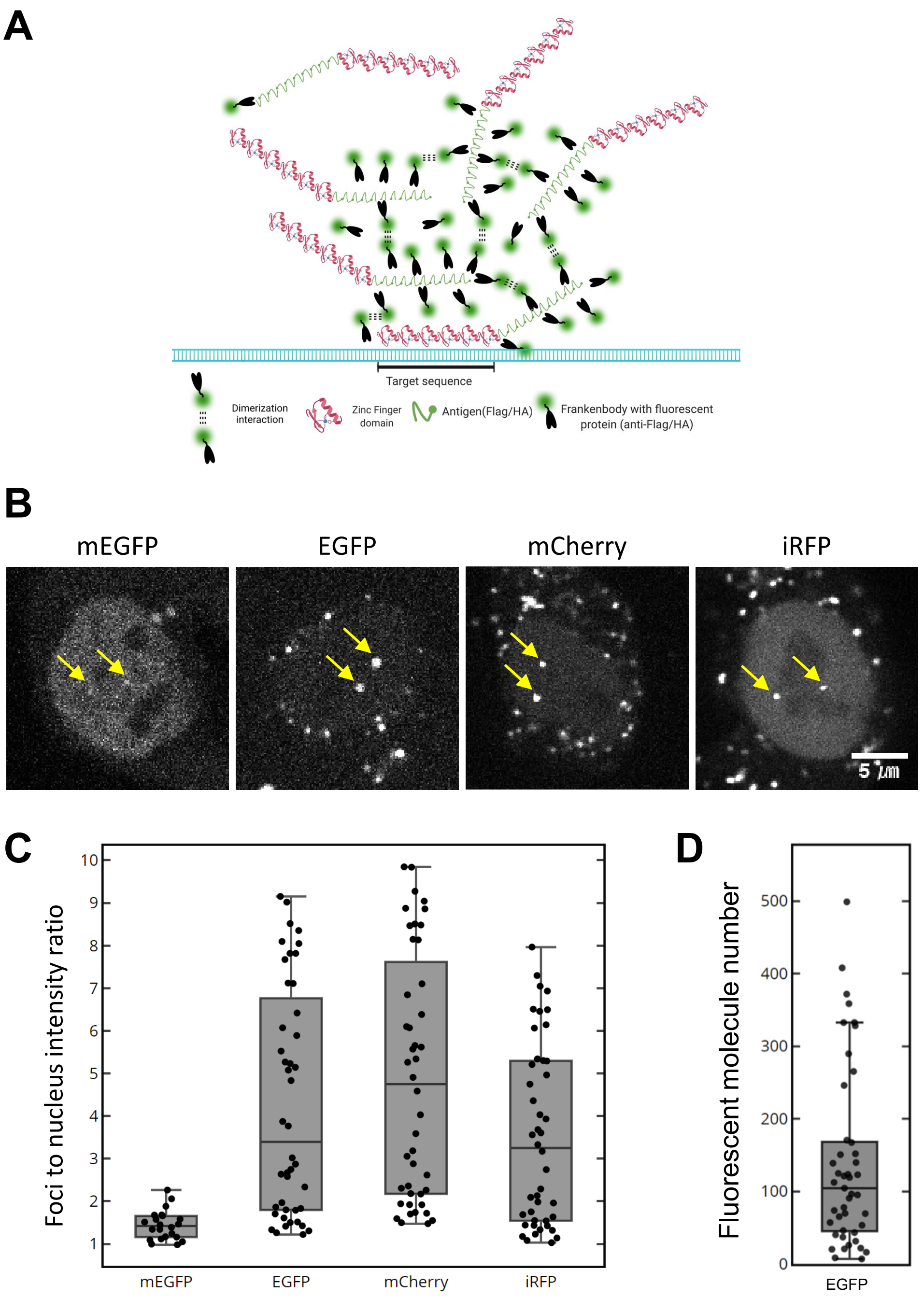

Fig. S4. Multimer formation and ZF probes with frankenbodies.
(A) A schematic illustration for a model that ZF probes and frankenbodies make a big complex through dimerization of fluorescent proteins. Such a complex contains multiple DNA binding domains and fluorescent proteins. (B and C) Wild-type HCT-116 cells were transfected with expression vectors for anti-HA frankenbody tagged with a fluorescent protein (mEGFP, EGFP, mCherry, and iRFP) and then loaded with ZF-F probe that harbor 10x HA. (B) Representative confocal images of living HCT-116 cells transfected with anti-HA frankenbody. Nuclear foci are indicated by yellow arrows. (C) Intensity ratios of foci to nuclear background of anti-HA frankenbodies tagged with a different fluorescent protein (N_{mEGFP} = 23, N_{EGFP} = 44, $N_{mCherry}$ = 45 and N_{iRFP} = 44; each from 2 dishes). See Supplementary Table S3. (D) Relative intensity of foci to that of single molecule fluorescence. The center line shows the median; the box indicates 25-75%; whiskers extend 1.5 times the interquartile range from the 25th and 75th percentiles; and data points are plotted. The average is 138 (N = 45). See Supplementary Table S4.

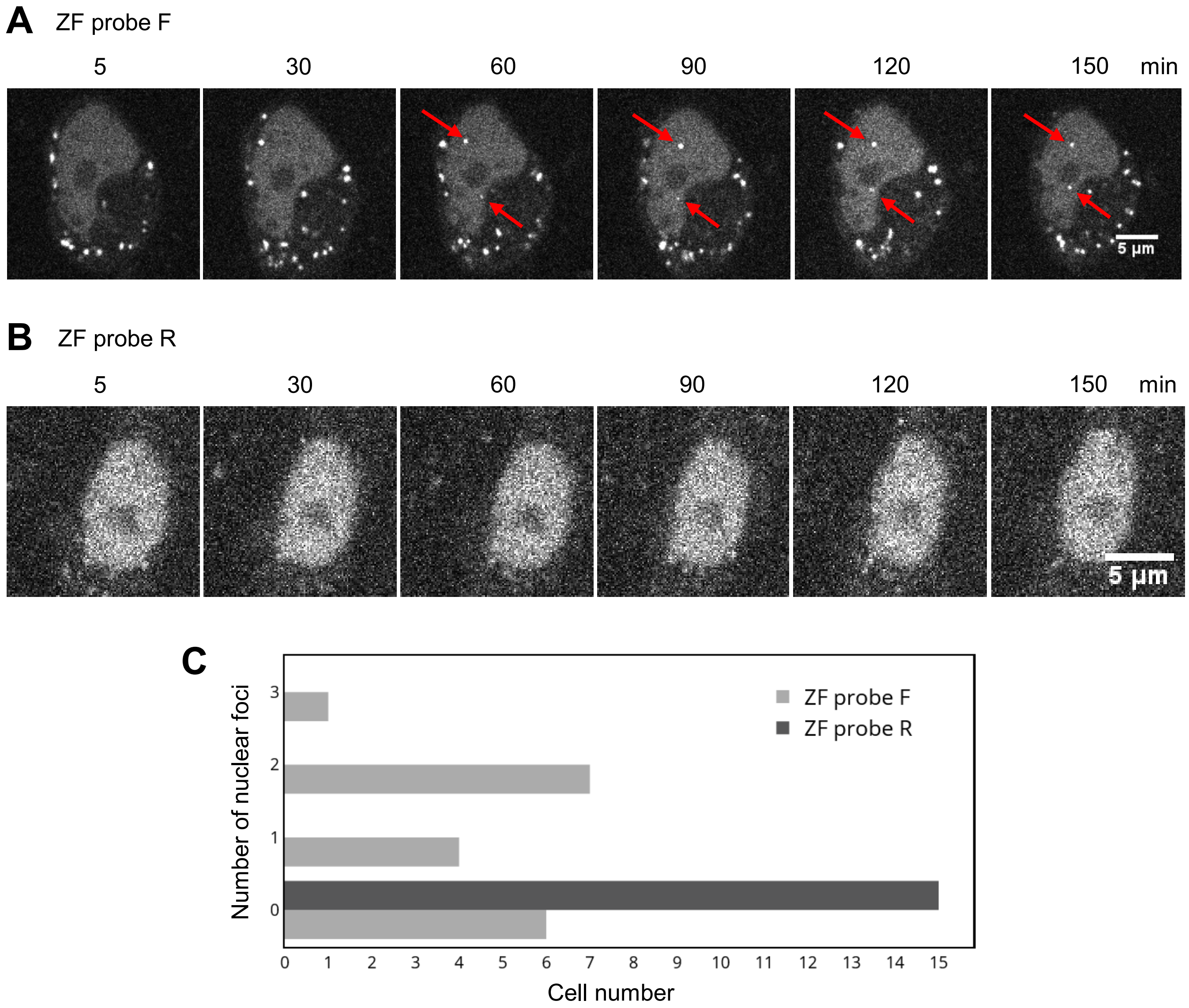

Fig. S5. ZF probes in mouse cells.

Mouse A9 cells were transfected with anti-HA mCherry or anti-FLAG iRFP frankenbody. After administrating ZF probes, time-lapse confocal images were collected. In the mouse genome, the target sequence of ZF probe F, but not R, is present. (A) ZF probe F with anti-HA mCherry-frankenbody. Nuclear foci are indicated by arrows. (B) ZF probe R with anti-FLAG iRFP-frankenbody. No nuclear foci were observed. (C) Number of nuclear foci per cell (n=18 and 15 for ZF probe F and R, respectively).

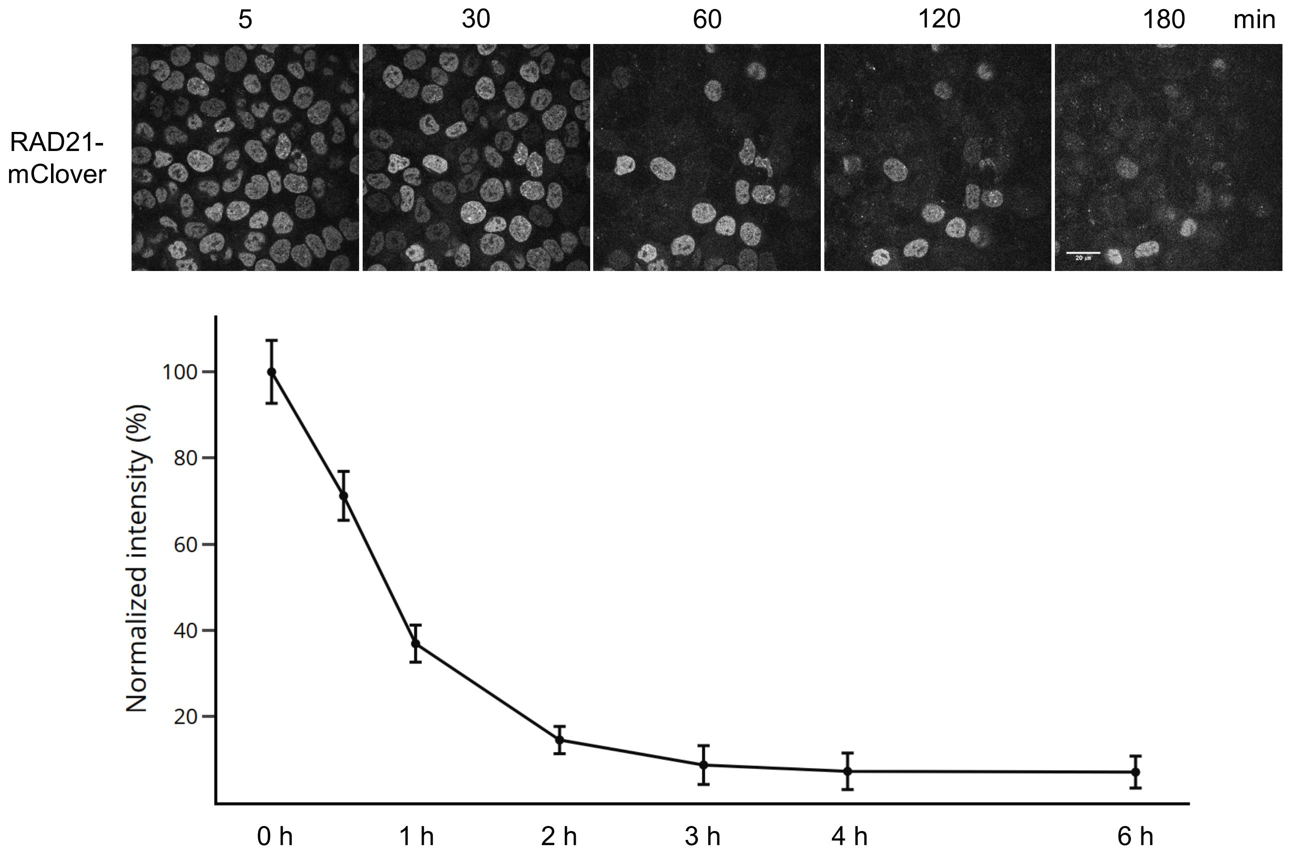

Fig. S6. Degradation of RAD21-mClover by auxin treatment.
After the addition of auxin in the medium, time-lapse confocal sections of HCT116 -RAD21-mAID-mClover cells were acquired. (top) Representative images of RAD21-mClover. (bottom) Normalized intensity of RAD21-mClover to that of time point 0 h (average ± SD; n=1024 pixel-points; from duplicate experiments).

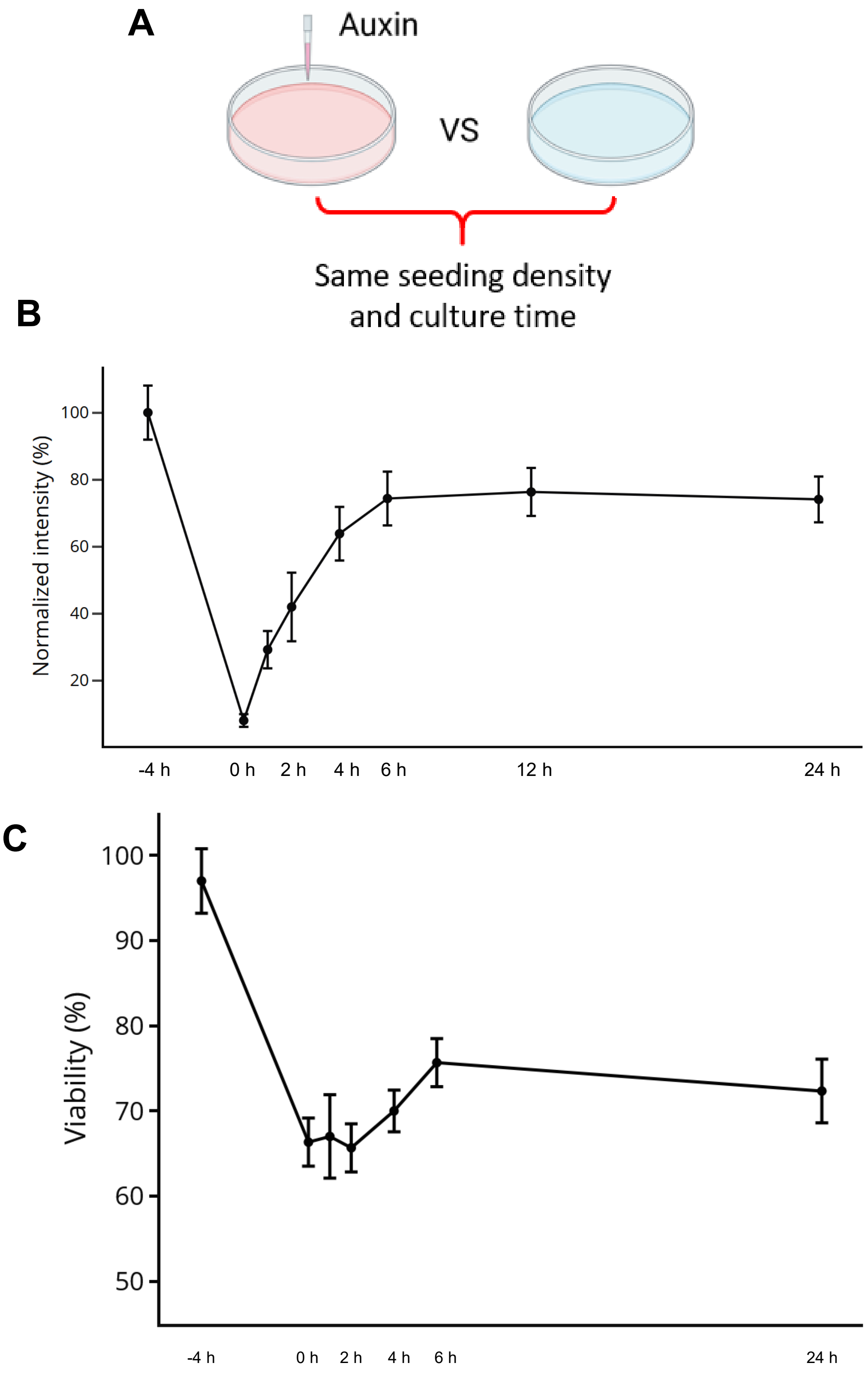

Fig. S7. Recovery of HCT116-RAD21-mAID-mClover cells after removal of auxin.
HCT116-RAD21-mAID-mClover cells were transfected with anti-FLAG and anti-HA frankenbody expression vectors, loaded with ZF probes, and treated with auxin for 4 h to degrade RAD21. After washing away auxin, cells were set on to a confocal microscope and further incubated with auxin-free medium. (**A**) A schematic illustration for how to seeding negative control (NC) sample for both RAD21-mClover-GFP intensity and cell viability. (**B**) Normalized RAD21-mClover-GFP intensity (average ± SD; n=107 cells). (**C**) Cell viability. Cell numbers were counted after the auxin removal (average ± SD; n=3 independent experiments).
